# Acute kidney injury following major emergency abdominal surgery – a retrospective cohort study based on medical records data

**DOI:** 10.1186/s12882-022-02708-8

**Published:** 2022-03-05

**Authors:** Theis B. Mikkelsen, Anders Schack, Jakob O. Oreskov, Ismail Gögenur, Jakob Burcharth, Sarah Ekeloef

**Affiliations:** Center for Surgical Science, Department of Surgery, Zealand University Hospital, Lykkebaekvej 1, 4600 Koege, Denmark

**Keywords:** Abdominal surgery, Acute kidney injury, Intensive care, Postoperative complications, Emergency surgery

## Abstract

**Background:**

Acute Kidney Injury (AKI) is a frequent and serious postoperative complication in trauma or critically ill patients in the intensive care unit. We aimed to estimate the risk of AKI following major emergency abdominal surgery and the association between AKI and 90-day postoperative mortality.

**Methods:**

In this retrospective cohort study, we included patients undergoing major emergency abdominal surgery at the Department of Surgery, Zealand University Hospital, Denmark, from 2010 to 2016. The primary outcome was the occurrence of AKI within postoperative day seven (POD7). AKI was defined according to the Kidney Disease: Improving Global Outcomes (KDIGO)-criteria. The risk of AKI was analysed with a multivariable logistic regression. The association between AKI and 90-day mortality was analysed with a multivariable survival analysis.

**Results:**

In the cohort, 122 out of 703 (17.4%) surgical patients had AKI within POD7. Of these, 82 (67.2%) had AKI stage 1, 26 (21.3%) had AKI stage 2, and 14 (11.5%) had AKI stage 3. Fifty-eight percent of the patients who developed postoperative AKI did so within the first 24 h of surgery. Ninety-day mortality was significantly higher in patients with AKI compared with patients without AKI (41/122 (33.6%) versus 40/581 (6.9%), adjusted hazard ratio 4.45 (95% confidence interval 2.69–7.39, *P* < 0.0001)), and rose with increasing KDIGO stage. Pre-existing hypertension and intraoperative peritoneal contamination were independently associated with the risk of AKI.

**Conclusions:**

The risk of AKI is high after major emergency abdominal surgery and is independently associated with the risk of death within 90 days of surgery.

**Supplementary Information:**

The online version contains supplementary material available at 10.1186/s12882-022-02708-8.

## Background

Acute kidney injury (AKI) is a frequent but often overlooked complication after major surgery [1–3]. The incidence ranges from 22 to 40% depending on the population and type of surgery [4–6]. Postoperative AKI has been found to increase the risk of subsequent morbidity and mortality in patients undergoing major elective surgery and in critically ill patients in the intensive care unit [1, 2]. The pathophysiology of AKI in relation to surgery is not fully understood, making it challenging to prevent. Ischemia, inflammation, oxidative stress, and genetics might be central factors included in the pathophysiology of postoperative AKI [7, 8]. Clinical risk factors include pre-existing chronic kidney disease, diabetes, advanced age and hypertension [2, 5, 6, 9]. A number of different systems for classification and diagnosis of AKI have been introduced over the years. Today, the predominant method is the Kidney Disease: Improving Global Outcomes (KDIGO)-criteria [10], where AKI is defined as an abrupt decrease in renal function and AKI can clinically be identified as a rise in s-creatinine levels and decreasing urine outputs [11].

The risk of postoperative complications and subsequent morbidity is high in patients undergoing major emergency abdominal surgery [12, 13]. Several organs are affected by the underlying pathology and the surgical stress response leading to an increased risk of perioperative single and multiorgan dysfunction [14]. Therefore, national and regional initiatives have in recent years been implemented in order to improve the clinical outcomes for this specific surgical population [15–17]. The risk and consequences of AKI are well known after major elective surgery and in critically ill surgical patients in the intensive care unit, however, no specific focus has been on AKI after major emergency abdominal surgery where the majority of the patients are treated in the general surgical ward postoperatively.

The aim of this retrospective cohort study was to estimate the risk of AKI following major emergency abdominal surgery and evaluate whether AKI is associated with 90-day postoperative mortality after emergency surgery.

## Methods

### Study design and setting

We conducted a retrospective cohort study reported according to The Strengthening the Reporting of Observational Studies in Epidemiology (STROBE) Statement [18]. We included patients who underwent major emergency abdominal surgery at Department of Surgery, Zealand University Hospital (Denmark) from January 2010 through September 2016.

The study was approved by the Danish Data Protection Agency (approval: REG-010–2017). In Denmark, a written informed consent and ethical approval are by law not necessary for retrospective database studies. Authors had full access to all data.

### Study population

All patients from the age of 18 undergoing major emergency (within 72 h of admission) abdominal surgery were included. Surgical procedures involving the stomach, small or large bowel, or rectum for conditions such as perforation, ischaemia, abdominal abscess, bleeding or obstruction, washout/evacuation of intra-peritoneal haematoma or abscess, laparotomy/laparoscopy with inoperable pathology (e.g., peritoneal/hepatic metastases), adhesiolysis, fascial dehiscence or any reoperation meeting the criteria above were included. Laparoscopic as well as open procedures were included if adherent to procedure-criteria. Patients who subsequently underwent several surgical procedures were eligible for inclusion if the primary procedure was includable. Patients who underwent minor abdominal procedures (appendectomy and cholecystectomy) as the sole surgical procedure were excluded. Trauma surgery was excluded due to the different pathophysiology. End-stage renal disease with dialysis or kidney transplantation were not exclusion criteria.

AKI was defined according to the KDIGO criteria [10]. The baseline s-creatinine level was defined as the s-creatinine level at admission. If multiple preoperative values were available from admission to the day of surgery, the highest value was chosen as baseline. Patients with no pre- or postoperative creatinine value were excluded. Postoperatively, patients were transferred to the standard surgical ward. However, patients in need of respiratory or cardiovascular support were transferred directly to the ICU. This decision was made by the attending anesthesiologist and/or surgeon.

As a standard, all patients received intravenous piperacillin-tazobactam on postoperative day one to three. Only patients that were admitted to the intensive care unit postoperatively were treated with vasoactive drugs.

### Data collection

Retrospectively, patients were included using NOMESCO-based procedure codes (Additional file 1). All patient data were collected from individual electronic patient records. This included: patient demographics, pre-, intra- and postoperative data, American Association of Anaesthesiologists classification (ASA-classification), WHO performance score, quick Sequential Organ Failure Assessment (qSOFA) at admission, and biochemical data (s-creatinine, estimated glomerular filtration rate (eGFR), blood urea, p-haemoglobin, p-sodium, p-potassium). The investigators calculated the Charlson Comorbidity Index (CCI) [19] based on ICD-codes available in the electronic patient records. The investigators evaluated the surgical complications by Clavien-Dindo score.

In Denmark, the vital status of all citizens is registered in the Danish Civil Registration System. This national registry is automatically linked with the electronic patient record. Data on 90-day mortality were extracted from the electronic patient record.

### Outcomes

The primary outcome was the incidence of postoperative AKI within seven days of surgery. Furthermore, the level of AKI was staged from 1 to 3. Postoperative AKI Stage 1 was according to the KDIGO criteria [10] defined as a 1.5–1.9-fold increase in s-creatinine level from baseline or an absolute 26.5 µmol l^−1^ increase in s-creatinine level over a period of 48 h or less within seven days of surgery. AKI Stage 2 was defined as a 2.0–2.9-fold increase in postoperative s-creatinine, and AKI Stage 3 as a ≥ threefold increase of postoperative s-creatinine from baseline or an absolute increase in s-creatinine to ≥ 353.6 µmol l^−1^, both over a period of 48 h or less within seven days of surgery. The binary primary outcome, postoperative AKI, was defined by the first elevated s-creatinine according to the KDIGO definition over a period of 48 h or less within seven days of surgery, whereas the grading of AKI (1–3) was based on the largest increase in s-creatinine over a period of 48-h or less within seven days of surgery. Secondary outcomes included 90-day mortality, intensive care unit (ICU) admission, lengths of stay, and surgical complications defined as a Clavien-Dindo score ≥ 3 [20].

### Statistical analysis

The population was stratified into patients with and without AKI (≥ KDIGO stage 1). Data distribution was examined with histograms and QQ-plots. Continuous data were presented as means with standard deviation (SD) or medians with interquartile range (IQR). Categorical data were presented as proportions and percentages. Categorical data were analysed using χ [2]-test or Fischer’s exact test as appropriate. Continuous data were analysed using student’s unpaired t-test or the Mann–Whitney U test. The difference in s-creatinine over time stratified on AKI was analysed with a mixed model with an unstructured variance–covariance structure. We performed a uni- and multivariable logistic regression by forced entry method to identify variables associated with the risk of postoperative AKI. Predefined independent variables included age group, sex, performance score, congestive heart failure, baseline s-creatinine, hypertension, diabetes, peritoneal contamination, intraoperative blood loss, and qSOFA. The variables were selected on the basis of clinical hypotheses and previous studies on postoperative AKI [21–23]. Results were expressed as unadjusted and adjusted odds ratios (OR) with 95% confidence interval (95% CI). The fitting of the model was tested with the Hosmer and Lemeshow Goodness of Fit test. Uni- and multivariable survival analysis on the association between AKI and 90-day postoperative mortality was performed. Predefined variables included in the analysis: AKI, baseline s-creatinine, age group, sex, CCI, type of surgical procedure, peritoneal contamination, and qSOFA. The model was adjusted for potential immortal time bias. The survival analysis fulfilled the proportional hazards assumption. Results were expressed as unadjusted and adjusted hazard ratios (HR) with 95% CI. In the multivariable logistic regression and the multivariable survival analysis, a maximum of 1 variable per 10 events were included to avoid overfitting of the model. Statistics were performed in SPSS statistical Software version 25. IBM. USA and SAS version 9.4 (SAS institute, USA). A two-sided *P*-value < 0.05 was considered statistically significant.

## Results

**Fig. 1:**
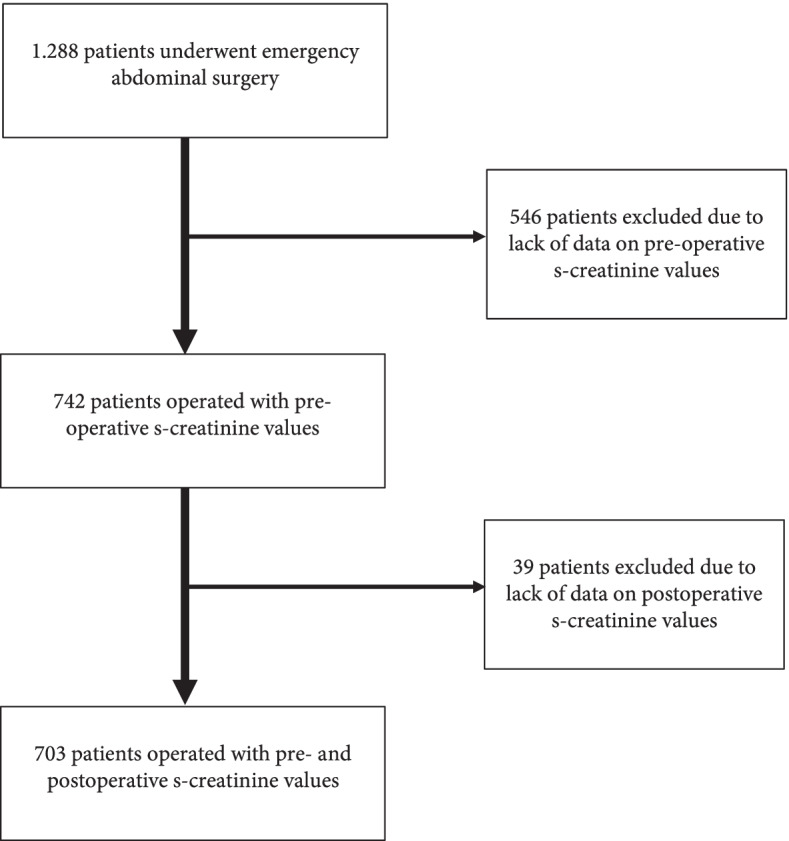
Patient flow

1,288 potentially eligible patients underwent major emergency abdominal surgery in the study period from January 2010 to September 2016. Of these, 546 patients were excluded since no baseline s-creatinine assessment was available and another 39 patients were excluded due to missing postoperative s-creatinine levels, leaving 703 eligible patients, Fig. 1.

Patient demographics and clinical characteristics are presented in Tables 1 and 2. One hundred and twenty-two of the 703 patients (17.4%) suffered AKI within postoperative day seven. Patients with AKI were significantly older, were more likely to be smoking, presented with lower performance scores and a higher ASA class and CCI. Looking at separate comorbidities, patients with AKI were more likely to suffer from hypertension and diabetes, Table 1. Patients with AKI had a higher qSOFA score at admission. Significantly more patients with AKI had intraoperative peritoneal contamination compared with patients without AKI (48.4% (59/122) versus 35.0% (203/581), *P* < 0.0001).

**Table 1:** Demographical characteristics

	**Total (** ***n*** ** = 703)**	**Patients without acute kidney injury** ^**a**^ ** (** ***n*** ** = 581)**	**Patients with acute kidney injury** ^**a**^ ** (** ***n*** ** = 122)**	***P*** **-value (acute kidney injury** ^**a**^ ** versus no acute kidney injury** ^**a**^ **)**
**Age (years)**				< 0.0001
60	240 (34.1%)	224 (38.6%)	16 (13.1%)	
> 60–70	176 (25.0%)	138 (23.8%)	38 (31.1%)	
> 70–80	160 (22.8%)	128 (22.0%)	32 (26.2%)	
> 80	127 (18.1%)	91 (15.7%)	36 (29.5%)	
**Female**	384 (54.6%)	319 (54.9%)	65 (53.3%)	0.74
**Active smoking**	144 (21.1%)	109 (19.3%)	35 (29.7%)	0.01
Missing	20 (2.8%)	16 (2.8%)	4 (3.3%)	
**Excessive alcohol use** ^**b**^	71 (10.1%)	59 (10.2%)	12 (9.8%)	0.92
**ASA** ^**c**^				0.004
I-II	464 (70.9%)	397 (73.2%)	67 (59.8%)	
≥ III	190 (29.1%)	145 (26.8%)	45 (40.2%)	
Missing	49 (7.0%)	39 (6.7%)	10 (8.2%)	
**Performance score**				0.03
0	487 (72.0%)	412 (74.2%)	75 (62.0%)	
1	143 (21.2%)	108 (19.5%)	35 (28.9%)	
≥ 2	46 (6.8%)	35 (6.3%)	11 (9.1%)	
Missing	27 (3.8%)	26 (4.5%)	1 (0.8%)	
**qSOFA** ^**d**^				0.02
0	258 (43.3%)	227 (45.6%)	31 (31.6%)	
1	284 (47.7%)	231 (46.4%)	53 (54.1%)	
≥ 2	54 (9.1%)	40 (8.0%)	14 (14.3%)	
Missing	107 (15.2%)	83 (14.3%)	24 (19.7%)	
**Chronic Kidney Disease**	30 (4.3%)	25 (4.3%)	5 (4.1%)	0.92
**Hypertension**	314 (44.7%)	228 (39.2%)	86 (70.5%)	< 0.0001
**Diabetes**	74 (10.5%)	53 (9.1%)	21 (17.2%)	0.008
**Ischaemic Heart Disease**	72 (10.2%)	55 (9.5%)	17 (13.9%)	0.14
**Heart Failure**	34 (4.8%)	26 (4.5%)	8 (6.6%)	0.33
**COPD** ^**f**^	104 (14.8%)	85 (14.6%)	19 (15.6%)	0.79
**Cerebrovascular Disease**	53 (7.5%)	44 (7.6%)	9 (7.4%)	0.94
**Charlson Comorbidity Index**				< 0.0001
0	324 (46.1%)	279 (48.0%)	45 (36.9%)	
1–2	270 (38.4%)	226 (38.9%)	44 (36.1%)	
> 2	109 (15.5%)	76 (13.1%)	33 (27%)	

Data are units (percentages) or mean (standard deviation) unless otherwise indicated

^a^Acute Kidney Injury was defined according to the KDIGO criteria

^b^Excessive alcohol use was defined as > 7 units of alcohol for women per week and > 14 unites of alcohol for men per week

*Abbreviations*: ^c^ *ASA* The American Society of Anaesthesiologists physical status classification; ^d^ *qSOFA* quick Sepsis Related Organ Failure Assessment, ^e^ *eGFR* estimated Glomerular Filtration Rate, *COPD* ^f^Chronic Obstructive Pulmonary Disease

**Table 2:** Perioperative characteristics

	**Total** **(** ***n*** ** = 703)**	**Patients without acute kidney injury** ^**a**^ **(** ***n*** ** = 581)**	**Patients with acute kidney injury** ^**a**^ **(** ***n*** ** = 122)**	***P*** **-value** **(acute kidney injury** ^**a**^ ** versus no acute kidney injury** ^**a**^ **)**
**Type of surgery**				0.31
Upper Gastrointestinal	45 (6.4%)	33 (5.7%)	12 (9.8%)	
Small intestine with resection	162 (23.0%)	129 (22.2%)	33 (27.0%)	
Colon with resection	109 (15.5%)	90 (15.5%)	19 (15.6%)	
Laparotomy	318 (45.2%)	268 (46.1%)	50 (41.0%)	
Combined small intestine and colon	50 (7.1%)	44 (7.6%)	6 (4.9%)	
Other	19 (2.7%)	17 (2.9%)	2 (1.6%)	
**Peritoneal contamination**				< 0.0001
None	440 (62.7%)	377 (65.0%)	63 (51.6%)	
Minimal	107 (15.2%)	91 (15.7%)	16 (13.1%)	
Local pus/abscess	60 (8.5%)	52 (9.0%)	8 (6.6%)	
Diffuse pus or faeces	95 (13.5%)	60 (10.3%)	35 (28.7%)	
**Peroperative blood loss**				0.84
< 100 ml	558 (88.7%)	454 (88.5%)	104 (89.7%)	
100-500 ml	49 (7.8%)	40 (7.8%)	9 (7.8%)	
> 500 ml	22 (3.5%)	19 (3.7%)	3 (2.6%)	
Missing	74 (10.5%)	68 (11.7%)	6 (4.9%)	
**Peroperative blood transfusion**	31 (4.4%)	23 (4.0%)	8 (6.6%)	0.19
**Transferred directly to the ICU postoperatively (on POD 0)**	53 (7.5%)	31 (5.3%)	22 (18.0%)	< 0.001
**s-Creatinine (µmol l** ^**−1**^ **),** Median (IQR)	79 (40)	77 (34)	93 (50)	< 0.0001
**eGFR [ml (min 1.73 m** ^**2**^ **)** ^**−1**^ **],** Median (IQR)^e^	65 (32)	68 (29.5)	58 (29.5)	< 0.0001
Missing	219 (31.2%)			
**Blood Urea (mmol l** ^**−1**^ **),** Median (IQR)	6.9 (5.1)	6.7 (4.8)	8.8 (6.1)	< 0.0001
Missing	61 (8.7%)			
**p-sodium (mmol l** ^**−1**^ **),** Median (IQR)	137 (5.0)	137 (5.0)	136 (6.0)	0.18
Missing	25 (3.6%)			
**p-potassium (mmol l** ^**−1**^ **),** Median (IQR)	3.7 (0.6)	3.7 (0.6)	3.6 (0.8)	0.26
Missing	28 (4.0%)			
**p-haemoglobin (mmol l** ^**−1**^ **),** Median (IQR)	8.5 (1.9)	8.5 (1.8)	8.4 (1.8)	0.16
Missing	35 (5.0%)			

Data are units (percentages) or mean (standard deviation) unless otherwise indicated

^a^Acute Kidney Injury was defined according to the KDIGO criteria. Biochemical parameters were assessed preoperatively

### Acute kidney injury and s-Creatinine changes

**Fig. 2:**
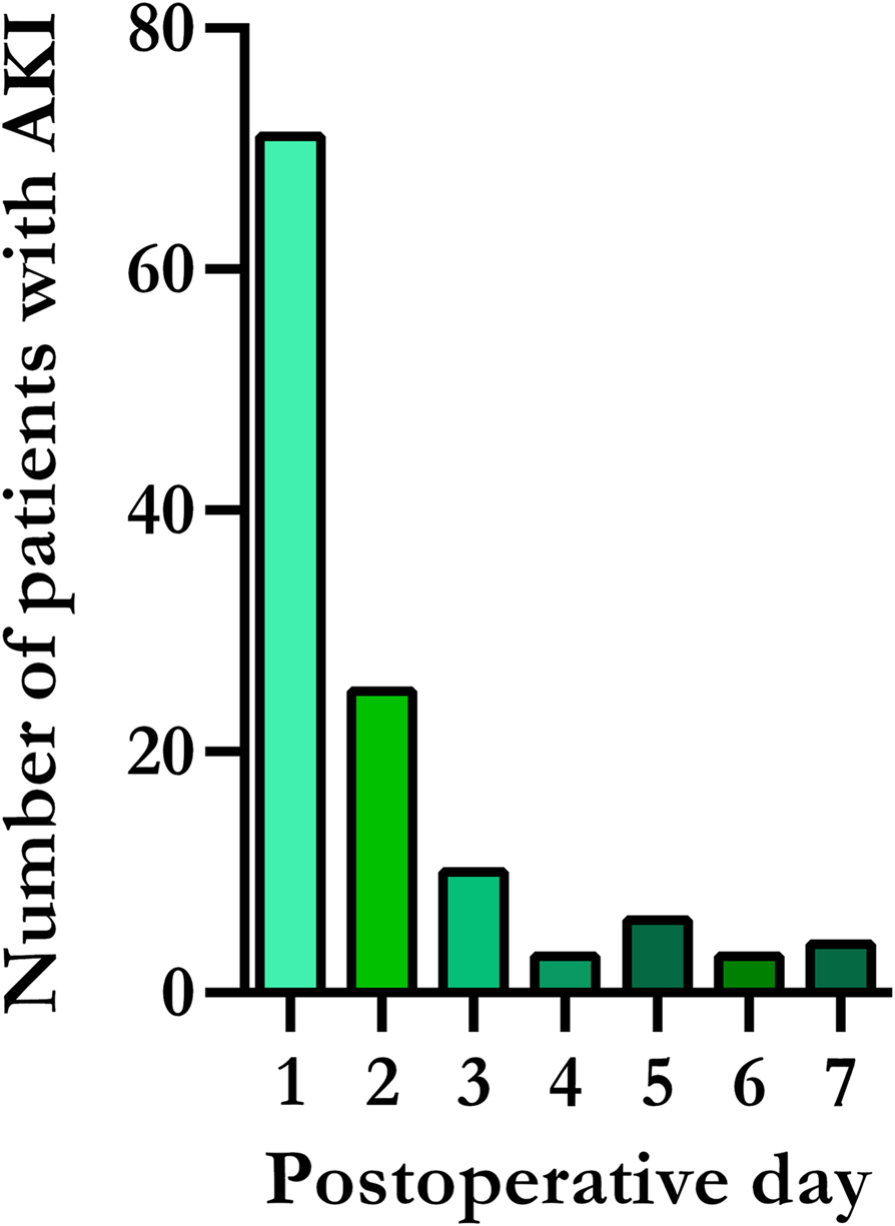
The occurrence of postoperative acute kidney injury*.* The figure illustrates on which postoperative day patients met the acute kidney injury criteria

**Fig. 3:**
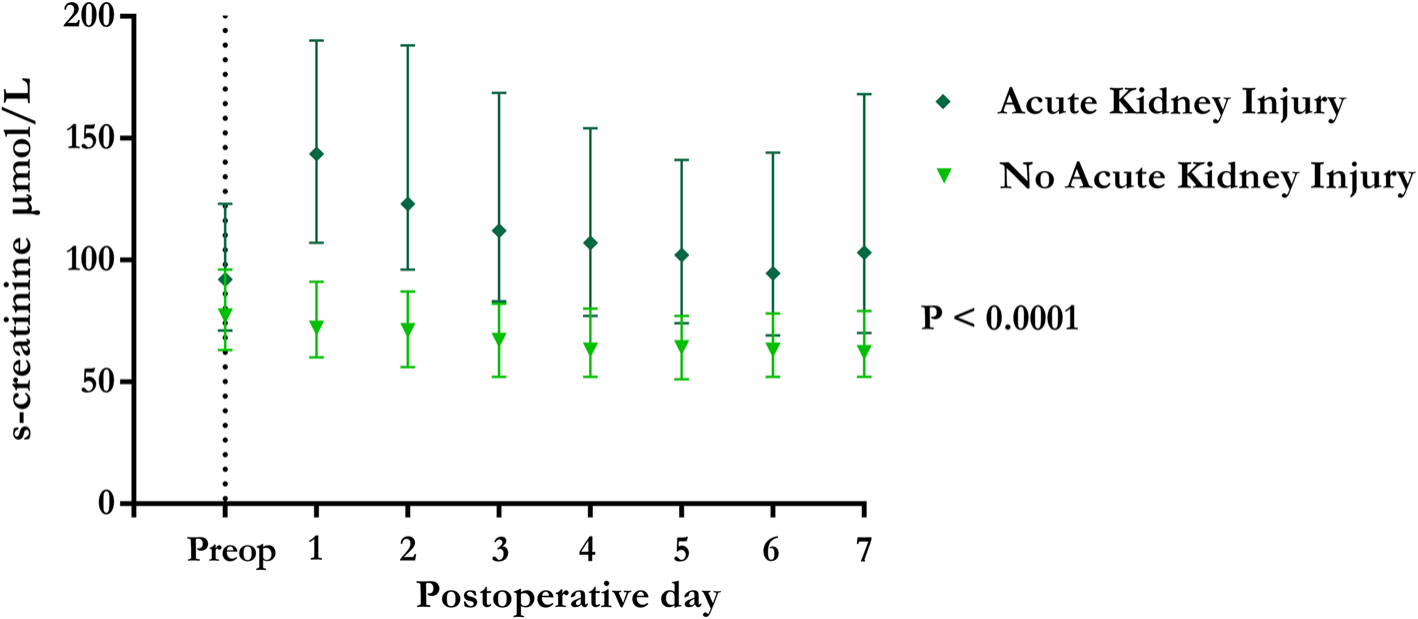
s-Creatinine level in the perioperative period in patients with and without acute kidney injury

Seventy-one of the 122 patients (58.2%) with postoperative AKI developed their kidney injury at postoperative day 1, Fig. 2. S-creatinine returned to baseline for 48.0% of the patients with AKI within postoperative day 7. Of the 122 patients that developed postoperative AKI, 82 (67.2%) had AKI stage 1, 26 (21.3%) had AKI stage 2, and 14 (11.5%) had AKI stage 3. Patients with AKI had a significantly higher s-creatinine during the postoperative period compared with patients without AKI, *P* < 0.0001, Fig. 3.

### Unadjusted clinical outcomes

**Fig. 4:**
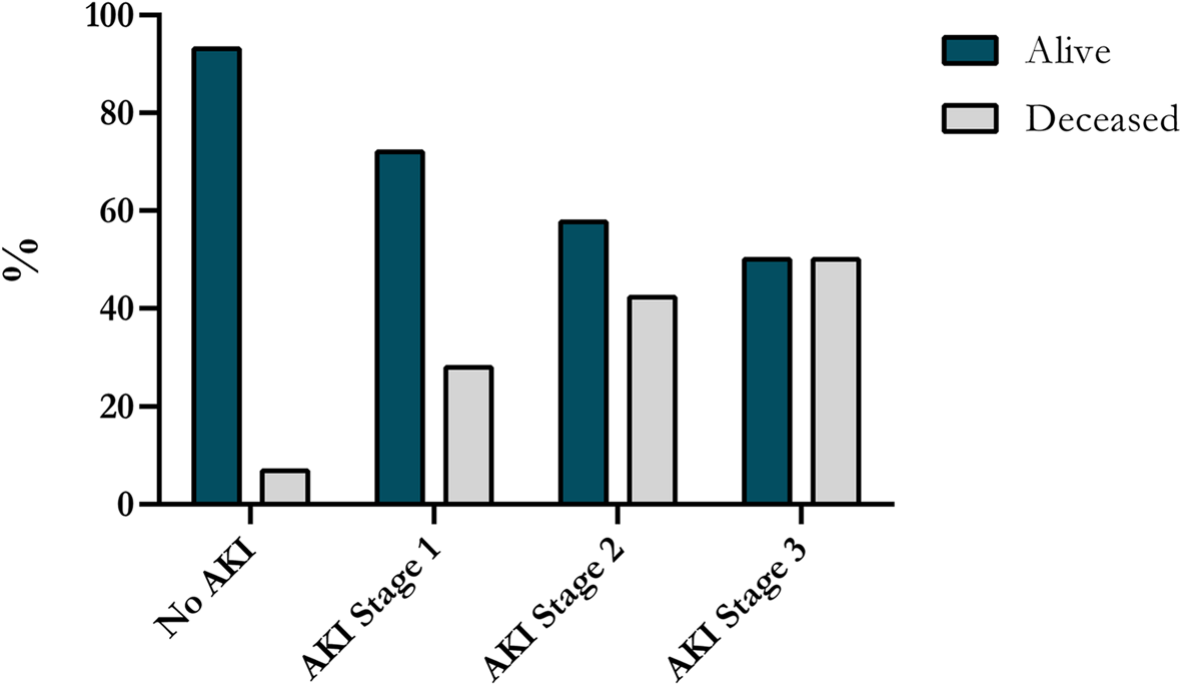
90-day mortality stratified on KDIGO stage

The overall 30-day and 90-day mortality were 8.7% (61/703 patients) and 11.5% (81/703 patients), respectively. Thirty-day and 90-day mortality were significantly higher in patients with postoperative AKI compared with patients without postoperative AKI (27.9% (34/122 patients) versus 4.7% (27/581 patients), *P* < 0.0001 and 33.6% (41/122 patients) versus 6.9% (40/581 patients), *P* < 0.0001, respectively). Moreover, 90-day mortality increased significantly with increasing KDIGO stage, *P* < 0.0001, as illustrated in Fig. 4.

More patients with AKI were admitted to the ICU compared with patients without AKI (36.1% (44/122 patients) versus 11.9% (69/581 patients), *P* < 0.0001). Stratified on KDIGO stage, 61.4% (27/44 patients) of the patients admitted to the ICU with AKI had AKI stage 1, 18.2% (8/44 patients) had AKI stage 2, and 20.4% (9/44 patients) had AKI stage 3, *P* = 0.064. Likewise, length of stay was significantly increased in patients with AKI compared with patients without AKI [17 days (IQR 10–27) versus 8 days (IQR 5–14), *P* < 0.0001]. Patients with AKI were more likely to suffer a surgical complication during hospital admission compared with patients without AKI (83/122 patients (68.0%) versus 166/581 patients (28.6%), *P* < 0.0001). All clinical outcomes stratified on KDIGO stages are presented in Table 3.

**Table 3:** Clinical outcomes stratified on KDIGO stage

	**Total** **(** ***n*** ** = 703)**	**No AKI** **(** ***n*** ** = 581)**	**AKI stage 1** **(** ***n*** ** = 82)**	**AKI stage 2** **(** ***n*** ** = 26)**	**AKI stage 3** **(** ***n*** ** = 14)**	***P-*** **value**
**90-day mortality**	81 (11.5%)	40 (6.9%)	23 (28.0%)	11 (42.3%)	7 (50%)	< 0.001
**Surgical complications**	132 (18.8%)	107 (18.4%)	19 (23.2%)	2 (7.7%)	4 (28.6%)	0.26
**Length of stay**, Median (IQR)	9 (11)	8 (9)	15 (18)	22 (15)	18 (8)	< 0.001
**ICU admission**	113 (16.1%)	69 (11.9%)	27 (32.9%)	8 (30.8%)	9 (64.3%)	< 0.001

Data are units (percentages) or mean (standard deviation) unless otherwise indicated

### Adjusted clinical outcomes

In the multivariable logistic regression, pre-existing hypertension and intraoperative peritoneal contamination were independently associated with the risk of postoperative AKI, Table 4.

**Table 4:** Uni- and multivariable logistic regression on the risk of acute kidney injury

	**Unadjusted OR** **(95% CI)**	***P*** **-value**	**Adjusted OR** **(95% CI)**	***P*** **-value**
**Baseline s-creatinine, µmol l** ^**−1**^	1.97 (1.33–2.90)	0.0007	1.35 (0.81- 2.25)	0.25
**Age group**		< 0.0001		0.10
< 60 years	1.00 (ref)		1.00 (ref)	
> 60–70 years	3.86 (2.07–7.18)		2.18 (1.00–4.75)	
> 70–80 years	3.50 (1.85–6.63)		1.99 (0.88–4.47)	
> 80 years	5.54 (2.93–10.48)		2.87 (1.22–6.72)	
**Female**	0.94 (0.63–1.39)	0.74	1.04 (0.61–1.78)	0.89
**Performance score**		0.03		0.70
0	1.00 (ref)		1.00 (ref)	
1	1.78 (1.13–2.80)		1.24 (0.67–2.29)	
≥ 2	1.73 (0.84–3.55)		1.41 (0.51–3.85)	
**Heart Failure**	1.50 (0.66–3.40)	0.33	0.70 (0.23–2.12)	0.53
**Hypertension**	3.70 (2.42–5.65)	< 0.0001	2.90 (1.65–5.10)	0.0002
**Diabetes**	2.07 (1.20–3.58)	0.009	1.81 (0.89–3.69)	0.10
**Peritoneal Contamination**		< 0.0001		0.004
None	1.00 (ref)		1.00 (ref)	
Minimal	1.05 (0.58–1.91)		1.02 (0.50–2.09)	
Local pus/abscess	0.92 (0.42–2.03)		0.78 (0.29–2.10)	
Diffuse pus or faeces	3.49 (2.13–5.73)		2.99 (1.58–5.64)	
**Peroperative blood loss**		0.71		0.55
< 100 ml	1.00 (ref)		1.00 (ref)	
100-500 ml	1.07 (0.52–2.20)		0.90 (0.37–2.19)	
> 500 ml	0.55 (0.12–2.42)		0.30 (0.03–2.70)	
**qSOFA** ^**a**^		0.02		0.51
0	1.00 (ref)		1.00 (ref)	
1	1.68 (1.04–2.71)		1.36 (0.76–2.42)	
≥ 2	2.56 (1.25–5.24)		1.49 (0.65–3.41)	

*95% CI* 95% confidence interval, *OR* odds ratio, ^a^ *qSOFA* quick Sepsis Organ Failure Assessment. The multivariable logistic regression was adjusted for baseline s-creatinine, age, sex, performance score, heart failure, hypertension, diabetes, peritoneal contamination, peroperative blood loss and quick Sepsis Organ Failure Assessment score

Table 5 displays uni- and multivariable 90-day survival analyses. Postoperative AKI was significantly associated with an increased 90-day mortality, adjusted HR 4.45 (95% CI 2.69–7.39), *P* < 0.0001.

**Table 5:** 90-days uni- and multivariable survival analysis

	**Unadjusted HR** **(95% CI)**	***P*** **-value**	**Adjusted HR** **(95% CI)**	***P*** **-value**
**Acute Kidney Injury**	5.19 (3.35–8.03)	< 0.0001	4.45 (2.69–7.39)	< 0.0001
**Baseline s-creatinine, µmol l** ^**−1**^	2.77 (1.92- 3.98)	< 0.0001	2.20 (1.35–3.59)	0.002
**Age group**		< 0.0001		< 0.0001
< 60	1.00 (ref)		1.00 (ref)	
> 60–70	3.39 (1.32–8.74)		1.53 (0.52–4.49)	
> 70–80	8.55 (3.57–20.45)		6.23 (2.37–16.38)	
> 80	9.37 (3.88–22.63)		5.16 (1.92–13.89)	
**Female**	1.30 (0.83–2.03)	0.25	1.18 (0.72–1.95)	0.43
**Charlson Comorbidity Index**		< 0.0001		0.001
0	1.00 (ref)		1.00 (ref)	
1–2	2.88 (1.60–5.17)		2.64 (1.37–5.07)	
> 2	5.58 (3.02–10.31)		4.37 (2.17–8.81)	
**Type of Surgery**		0.06		0.10
Upper gastrointestinal	1.00 (ref)		1.00 (ref)	
Small intestine with resection	0.52 (0.25–1.07)		0.60 (0.27–1.31)	
Colon with resection	0.57 (0.26–1.24)		0.91 (0.37–2.27)	
Laparotomy (no resection)	0.34 (0.17–0.68)		0.39 (0.18–0.88)	
Combined small intestine and colon	0.48 (0.18–1.30)		1.29 (0.45–3.67)	
Other	0.21 (0.026–1.59)		0.44 (0.06–3.53)	
**Peritoneal Contamination**		0.002		0.35
None	1.00 (ref)		1.00 (ref)	
Minimal	1.45 (0.77–2.72)		1.35 (0.69–2.65)	
Local pus/abscess	1.83 (0.88–3.79)		1.63 (0.73–3.63)	
Diffuse pus or faeces	2.83 (1.67–4.80)		1.26 (0.621–2.54)	
**qSOFA** ^**a**^		0.04		0.18
0	1.00 (ref)		1.00 (ref)	
1	1.01 (0.60–1.70)		0.95 (0.56–1.62)	
≥ 2	2.28 (1.15–4.50)		1.90 (0.92–3.93)	

*95% CI* 95% confidence interval, *HR* Hazard ratio, *ref* reference level, ^a^qSOFA, quick Sepsis Organ Failure Assessment. The multivariable survival analysis was adjusted for acute kidney injury, baseline s-creatinine, age, sex, Charlson Comorbidity Index, type of surgery, peritoneal contamination, and quick Sepsis Organ Failure Assessment score

## Discussion

In this retrospective cohort study, we found that 122/703 (17.4%) of patients undergoing major emergency abdominal surgery developed AKI within seven days of surgery. AKI primarily occurred within 24 h of surgery and only one in two patients with AKI had a normalised s-creatinine level on day seven after surgery. AKI was independently associated with an increased risk of 90-day mortality. Increasing age, previous hypertension and intraoperative peritoneal contamination were independently associated with the risk of postoperative AKI.

In elective surgery, the incidence of AKI ranges from 5.3% to 6.3% [24, 25]. This is considerably lower than the incidence of AKI in our cohort of surgical high-risk patients. We found that the patients that developed AKI were older, had a higher s-creatinine and blood urea at admission, and more commonly suffered from hypertension and diabetes. These findings suggest that the patients that develop AKI after major emergency abdominal surgery already before surgery had an affected kidney function, however, not to a point where this became clinically significant. Furthermore, intraoperative intraperitoneal severe contamination was a potent risk factor for development of AKI. This points towards a septic and inflammatory component partaking. The pathophysiology of postoperative AKI is complex and multifactorial [26]. Major direct causes are believed to be kidney hypoperfusion, ischaemia and inflammation [25, 27], all of which may arise due to the surgically induced stress response and sepsis [28, 29]. Consequently, patients that developed AKI had a higher qSOFA score at admission indicating that sepsis may be one of the central players in this population. However, the observational nature of our study does not permit conclusions on cause-effect relationships.

Fluid balance is essential for the perfusion of the kidneys and is largely disturbed in patients with sepsis and abdominal pathology such as small bowel obstruction [30, 31]. Perioperative fluid, hemodynamic management and risk of AKI is a much-debated topic in perioperative medicine [32]. A large multicentre randomised clinical trial on restrictive versus liberal fluid therapy in major elective abdominal surgery found the incidence of AKI, renal replacement therapy and surgical site infection to be significantly reduced in the liberal fluid group compared with the restrictive [33]. Other studies have examined the use of per- and postoperative goal-directed fluid therapy, intraoperative standardised management of hypotension and targeted oliguria reversal as strategies to prevent AKI [34–36]. However, the effects of these strategies on preventing AKI are still being debated. In our cohort, the perioperative fluid administration was not standardised, and goal-directed fluid therapy was not applied.

In our study, we found that postoperative AKI was independently associated with overall 90-days mortality. This confirms findings from previous studies on patients undergoing non-cardiac surgery [2, 6]. It is then important to consider whether AKI is a cause of mortality or if AKI is a symptom and consequence of underlying severe acute illness leading to single and multiorgan dysfunction. We found that almost 60% of the patients that developed AKI did so within the first 24 h of surgery. AKI may potentially be used as an early marker of underlying acute systemic illness or surgical complications. The predictive value of AKI needs to be confirmed in future studies.

In our study, we found that only 48% of AKI-patients had normalised s-creatinine levels within seven days of surgery. It is therefore reasonable to consider whether short-term postoperative AKI could potentially lead to a chronic kidney injury [37]. A cohort study including 390 patients undergoing urgent or elective major non-vascular abdominal surgery found that 47% of the patients that developed postoperative AKI experienced long term adverse renal outcomes (*P* < 0.0001), defined as the need for long-term dialysis and/or a 25% decrease in eGFR after hospital discharge [38]. Thus, even if AKI is a consequence of underlying acute illness, it may also be associated with long term adverse outcomes.

### Strengths and limitations

The study is limited by its observational nature leaving a risk for residual confounding. Potential eligible patients were excluded due to missing baseline s-creatinine level. This could potentially have introduced a selection bias; however, we were not able to identify a systematic cause that could explain the missing data. Baseline s-creatinine was defined as the admission assessment. Potentially, the admission s-creatinine could already be elevated leaving a risk of underestimating the total (pre- and postoperative) occurrence of AKI. However, the focus of the study was to estimate the effect of the surgical procedure including the surgically induced stress-response on the risk of AKI, therefore, the difference in s-creatinine pre- versus post-surgery was the primary assessment of interest. We chose the highest preoperative s-creatinine value as baseline if multiple values were available to be sure not to include patients with a sole preoperative AKI. Patients were defined as having postoperative AKI if they met the KDIGO criteria postoperatively regardless of whether the damage started before or after surgery. We were unable to comment on whether a patient has AKI at admission. Moreover, we used the KDIGO Guidelines to identify cases of postoperative AKI in the cohort. However, we did not have access to urine output data, and we might have missed some cases of AKI. The study was limited by its single centre design and the results may not be generalisable. The results should be confirmed in a larger population including more centres. According to the electronic medical records, 4.3% of the population had chronic kidney disease. The prevalence of chronic kidney disease in other European countries were estimated to 5.4–6.2% and higher among elderly and individuals with hypertension, diabetes, or cardiovascular disease [39–41]. A substantial part of the patients in our study might have had undiagnosed chronic kidney disease since many were elderly with hypertension or diabetes. Therefore, chronic kidney disease as a risk factor for acute kidney injury cannot with certainty be assessed in this study. Another limitation was a lack of standardisation of the perioperative fluid administration in the cohort and the fact that goal-directed fluid therapy was not applied. Furthermore, we did not have data on intraoperative hypotension. The study was reported according to international guidelines and all analyses were predefined including the variables in the multivariable analyses.

## Conclusions

In conclusion, one in five patients had postoperative AKI after major emergency abdominal surgery and AKI occurred within few days of the surgical procedure. The presence of AKI significantly increased the risk of death within 90 days of surgery. The pathophysiology of AKI should be further studied to develop preventive perioperative strategies.

## Supplementary Information

**Additional file 1.**

## Data Availability

Authors had full access to all data. The datasets used and/or analysed during the current study are available from the corresponding author on reasonable request.

## Abbreviations

AKI: Acute Kidney Injury
POD7: Postoperative day seven
KDIGO: Kidney Disease: Improving Global Outcomes
STROBE: The Strengthening the Reporting of Observational Studies in Epidemiology ASA: American Association of Anaesthesiologists classification
qSOFA: Quick Sequential Organ Failure Assessment
CCI: Charlson Comorbidity Index
eGFR: Estimated glomerular filtration rate
ICU: Intensive care unit
SD: Standard deviation
IQR: Interquartile range
OR: Odds ratio
95% CI: 95% Confidence interval
HR: Hazard ratio
