# Supplementary material for: Acute kidney injury following major emergency abdominal surgery – a retrospective cohort study based on medical records data
Source: BMC Nephrol. 2022 Mar 5;23:94. doi: 10.1186/s12882-022-02708-8 (PMC8897898; doi:10.1186/s12882-022-02708-8)
Supplement: Supplementary file 1 — Additional file 1. [file 12882_2022_2708_MOESM1_ESM.docx]

**Appendix A** | NOMESCO-CODES INVESTIGATED

KJAH01, KJAH00, KJDA60, KJDA61, KJDC10, KJDC11, KJDC20, KJDH00, KJDH70, KJDH71, KJWC00, KJWE00, KJFB00, KJFB01, KJFB20, KJFB30, KJFB30A, KJFB31, KJFB33, KJFB40, KJFB43, KJFB46, KJFB50, KJFB60, KJFB61, KJFB63, KJFB96, KJFH10, KJFM00, KJFF10, KJFF13, KJFF26, KJFK00, KJFK01, KJFK10, KJFK20, KJFK96, KJFL10, KJFL20.
